# Competition and mimicry: the curious case of chaetae in brachiopods from the middle Cambrian Burgess Shale

**DOI:** 10.1186/s12862-015-0314-4

**Published:** 2015-03-13

**Authors:** Timothy P Topper, Luke C Strotz, Lars E Holmer, Zhifei Zhang, Noel N Tait, Jean-Bernard Caron

**Affiliations:** Department of Earth Sciences, Palaeobiology, Uppsala University, Villavägen 16, SE - 752 36 Uppsala, Sweden; Department of Palaeobiology, Swedish Museum of Natural History, P.O. Box 50007, , SE-104 05 Stockholm, Sweden; Department of Biological Sciences, Macquarie University, Sydney, NSW 2109 Australia; Early Life Institute and Department of Geology, State Key Laboratory for Continental Dynamics, Northwest University, Xian, 710069 China; Department of Natural History (Palaeobiology Section), Royal Ontario Museum, 100 Queen’s Park, Toronto, Ontario M5S2C6 Canada; Department of Ecology and Evolutionary Biology, University of Toronto, 25 Willcocks Street, Toronto, Ontario M5S3B2 Canada; Department of Earth Sciences, University of Toronto, 22 Russell Street, Toronto, Ontario M5S3B1 Canada

**Keywords:** *Micromitra*, *Paterina*, Lagerstätten, Batesian mimicry, Setae, Polychaete, Lophotrochozoa

## Abstract

**Background:**

One of the first phyla to acquire biomineralized skeletal elements in the Cambrian, brachiopods represent a vital component in unraveling the early evolution and relationships of the Lophotrochozoa. Critical to improving our understanding of lophotrochozoans is the origin, evolution and function of unbiomineralized morphological features, in particular features such as chaetae that are shared between brachiopods and other lophotrochozoans but are poorly understood and rarely preserved. *Micromitra burgessensis* and *Paterina zenobia* from the middle Cambrian Burgess Shale are among the most remarkable examples of fossilized chaetae-bearing brachiopods. The form, functional morphology, evolutionary and ecological significance of their chaetae are studied herein.

**Results:**

Like in Recent forms, the moveable but semi-rigid chaetae fringe both the dorsal and ventral mantle margins, but in terms of length, the chaetae of Burgess Shale taxa can exceed twice the maximum length of the shell from which it projects. This is unique amongst Recent and fossil brachiopod taxa and given their size, prominence and energy investment to the organism certainly had an important functional significance. *Micromitra burgessensis* individuals are preserved on hard skeletal elements, including conspecific shells, *Tubulella* and frequently on the spicules of the sponge *Pirania muricata*, providing direct evidence of an ecological association between two species. Morphological analysis and comparisons with fossil and extant brachiopod chaetae point to a number of potential functions, including sensory, defence, feeding, defouling, mimicry and spatial competition.

**Conclusions:**

Our study indicates that it is feasible to link chaetae length to the lack of suitable substrate in the Burgess Shale environment and the increased intraspecific competition associated with this. Our results however, also lend support to the elongated chaetae as an example of Batesian mimicry, of the unpalatable sponge *Pirania muricata*. We also cannot discount brachiopod chaetae acting as a sensory grille, extending the tactile sensitivity of the mantle into the environment, as an early warning system to approaching predators.

**Electronic supplementary material:**

The online version of this article (doi:10.1186/s12862-015-0314-4) contains supplementary material, which is available to authorized users.

## Background

Brachiopods are bivalved, lophophore-bearing lophotrochozoans that dominated the Palaeozoic benthic marine realm. With one of the most complete fossil records of any animal phylum [1], they were also one of the first organisms to acquire biomineralized skeletal elements [1-3] and represent a vital component in unraveling the early evolution and relationships of the lophotrochozoa. The majority of fossil brachiopod taxa are known exclusively from their shells, with the preservation of distinctive unbiomineralized parts (including chaetae) typically restricted to Lagerstätte deposits, such as the lower Cambrian Chengjiang Lagerstätte [4] and the Burgess Shale Lagerstätte [5,6]. The rarity of preserved brachiopod unbiomineralized parts in the fossil record has hindered discussions regarding their early evolution and ecology, and the early evolution of the lophotrochozoa generally, largely due to disagreements about the interpretation of structures relative to extant phyla [7] and the apparent loss and development of key characters across groups [8]. For example, molecular studies have placed the Brachiopoda within the Trochozoa, closely related to the Nemertea, despite lacking a trochophore larval stage and possessing chaetae identical to annelids [8]. Only recently the chaetae of annelids and brachiopods were considered to be a homologous character [7]. Documentation of exquisitely preserved brachiopods including unbiomineralized features from Cambrian Lagerstätten, gives us a valuable window into the origins and functions of significant features that hold an evolutionary and functional importance in extant lophotrochozoan taxa.

Bristle or hair-like chitinous structures that project from the epidermis or exoskeleton are present across a range of lophotrochozoan organisms. Referred to as chaetae (also setae in the literature), these cuticular projections perform a range of sensory and locomotory tasks and are seen as a key character in phylogenetic analyses [9-11]. Perhaps the most well-known cuticular projections are the chaetae of the lophotrochozoan polychaete annelids. Annelid chaetae display a spectacular array of morphologies and are an important discriminator for species determination and phylogenetic placement in modern and fossil forms [11-14]. As a result, the chaetae in annelids have been intensely studied [12,13,15]. The same cannot be said for brachiopod chaetae. Brachiopod chaetae, in both fossil and extant taxa, do not show the same morphological variation seen in annelid chaetae and tend to be more simple, tapering, pointed forms [16-19]. Because they have not undergone the same intense examination as annelid chaetae, there exists a plethora of questions regarding their form, function, evolution and phylogenetic significance.

Adult brachiopod chaetae emerge from follicles along the dorsal and ventral mantle margins and occur in nearly all extant brachiopod groups, with the exception of craniid, megathyridid and the enigmatic cementing thecideidines [19-25]. Brachiopod chaetae have been recorded in multiple fossil brachiopod clades and in the vast majority of extant taxa [4,26,27]. This suggests that the possession of chaetae is likely a ubiquitous character in the Brachiopoda. It is potentially an ancestral feature and, like for other groups of lophotrochozoans [12,13,19], holds both taxonomic and phylogenetic significance.

Studies of extant brachiopod chaetae have tended to focus on their ultrastructure, composition and development [16-19,22,23,28] or on their use as part of larger scale phylogenetic analyses [10,11,29-31]. Despite their apparent morphological simplicity, a range of functions have been proposed for brachiopod chaetae [19,21,32,33]. The bundles of chaetae present in the lecithotrophic larval stage of Rhynchonelloids or Terebratelloids may be used for defence [34,35], as part of a sensory complex [19,21] or as a buoyancy aid, hindering the larvae from sinking in the water column [19]. The chaetae in adult brachiopods have been suggested as functioning as sensory grilles [21,32], assisting in the creation of currents for feeding purposes [21,33], sieving of inhalant currents [24,36,37], protection [37], burrowing [38] and as a deterrent in competition for space in intertidal zones [34]. Many of these functional interpretations remain to be rigorously tested and none have taken fossil taxa into account, despite Recent brachiopods, in terms of diversity, representing only a small fraction of what was a much larger clade in the Palaeozoic [24].

**Figure 1:**
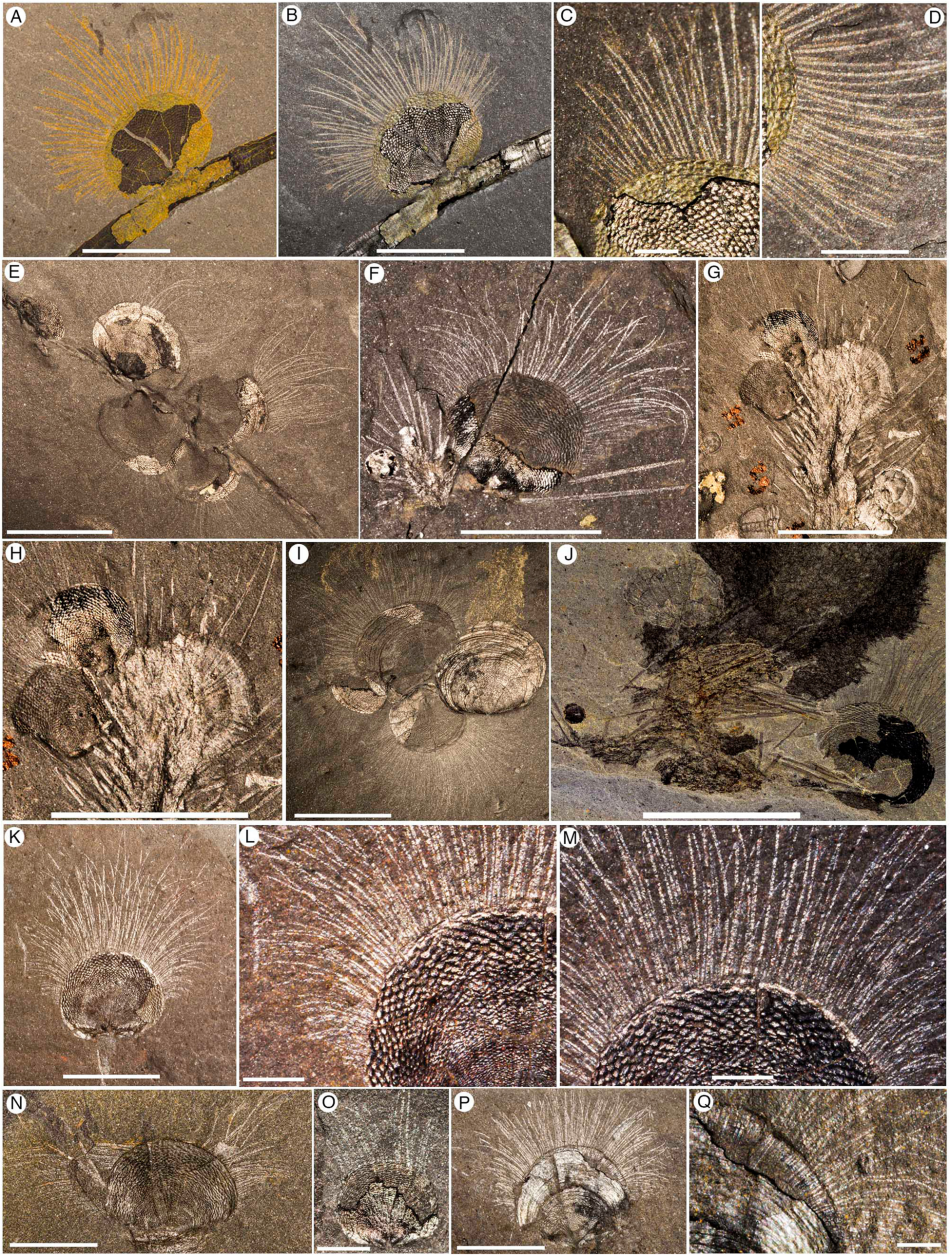
***Micromitra burgessensis*** **from the middle (Series 3, Stage 5) Cambrian Burgess Shale. A-D**, ROM63169, RQ + 8.2 m. **A-B**, Plan view of specimen attached to *Tubulella*; **C-D**, Close up anteromedial and posterolateral chaetae, scale bar 1 mm. **E**, ROM63170, RQ + 8.2 m, Plan view of specimens attached to *Tubulella*, scale bar 10 mm. **F**, USNM 59801a, Walcott Quarry, Phyllopod Bed, Plan view of *Acrothyra* and *Micromitra* attached to *Pirania*. **G-H**, ROM57839.30-33, BW-150 cm. **G**, Plan view of *Nisusia* and *Micromitra* attached to *Pirania*, scale bar 10 mm. **H**, Close up of specimens attached to *Pirania*, scale bar 10 mm. **I**, ROM63171, RQ + 11.4 m, Plan view of a cluster of *Micromitra* specimens, scale bar 10 mm. **J**, ROM56248, BW-100 cm, Plan view of *Micromitra*, *Nisusia* and *Acrothyra* attached to *Pirania*, scale bar 10 mm. **K-M**, USNM 59801a, Walcott Quarry, Phyllopod Bed. **K**, Plan view. **L**, Close up of posterolateral chaetae, scale bar 1 mm. **M**, Close up of anteromedial chaetae, scale bar 1 mm. **N**, ROM63172, BW-210 cm, *Micromitra* with swept chaetae. **O**, ROM63173, BW-210 cm, juvenile *Micromitra* specimen, scale bar 1 mm. **P-Q**, ROM63174, BW-235 cm. **P**, Plan view; **Q**, Close up of chaetae beneath shell, scale bar 1 mm. Scale bars 5 mm unless otherwise stated.

**Figure 2:**
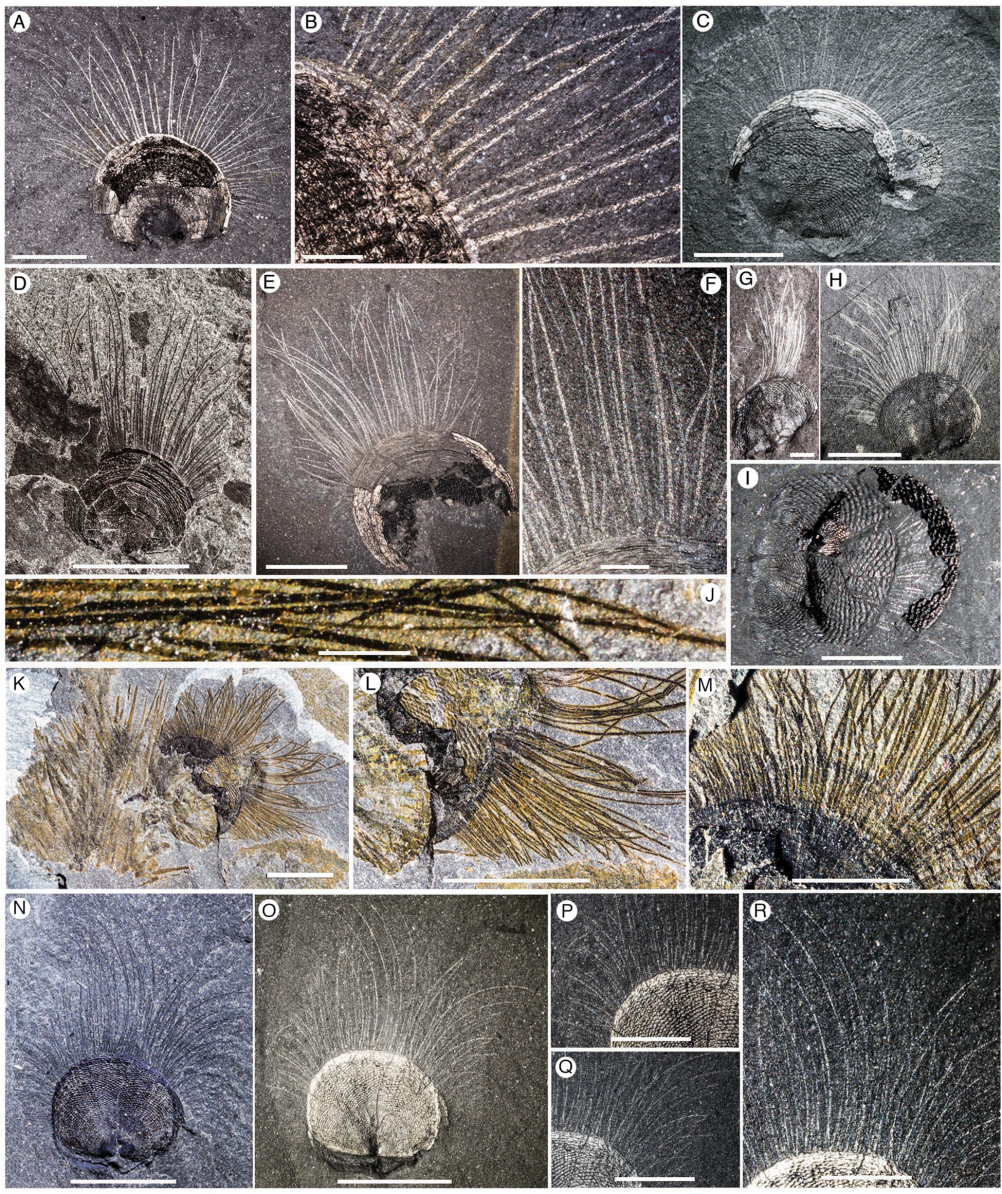
***Micromitra burgessensis*** **from the middle (Series 3, Stage 5) Cambrian Burgess Shale. A-B**, USNM 69646, Walcott Quarry, Phyllopod Bed. **A**, Plan view. **B**, Close up of anteromedial chaetae, scale bar 1 mm **C**, ROM63175, RQ + 8.1 m, Plan view. **D**, ROM57603, BW-120 cm, Plan view. **E-F**, ROM63176, RQ + 8.2 m. **E**, Plan view. **F**, Close up of anteromedial chaetae, scale bar 1 mm. **G**, ROM63177, BW-235 cm, *Micromitra* with swept chaetae, scale bar 1 mm. **H**, ROM63178, WT talus. Plan view. **I**, ROM57597, BW-235, Dissociated valves with chaetae preserved, scale bar 2.5 mm. **J-M**, ROM63180, WQ + 30. **J**, Close up of anteromedial chaetae, scale bar 1 mm. **K**, Plan view of *Micromitra* attached to *Pirania*. **L**, Close up of posterolateral chaetae. **M**, Close up of anteromedial chaetae, scale bar 2.5 mm. **N-R**, ROM63181, RQ + 8.4 m. **N-O**, Plan views. **P-Q**, Close up of chaetae, scale bars 2.5 mm. **R**, Close up of anteromedial chaetae, scale bars 2.5 mm. Scale bars 5 mm unless otherwise stated.

**Figure 3:**
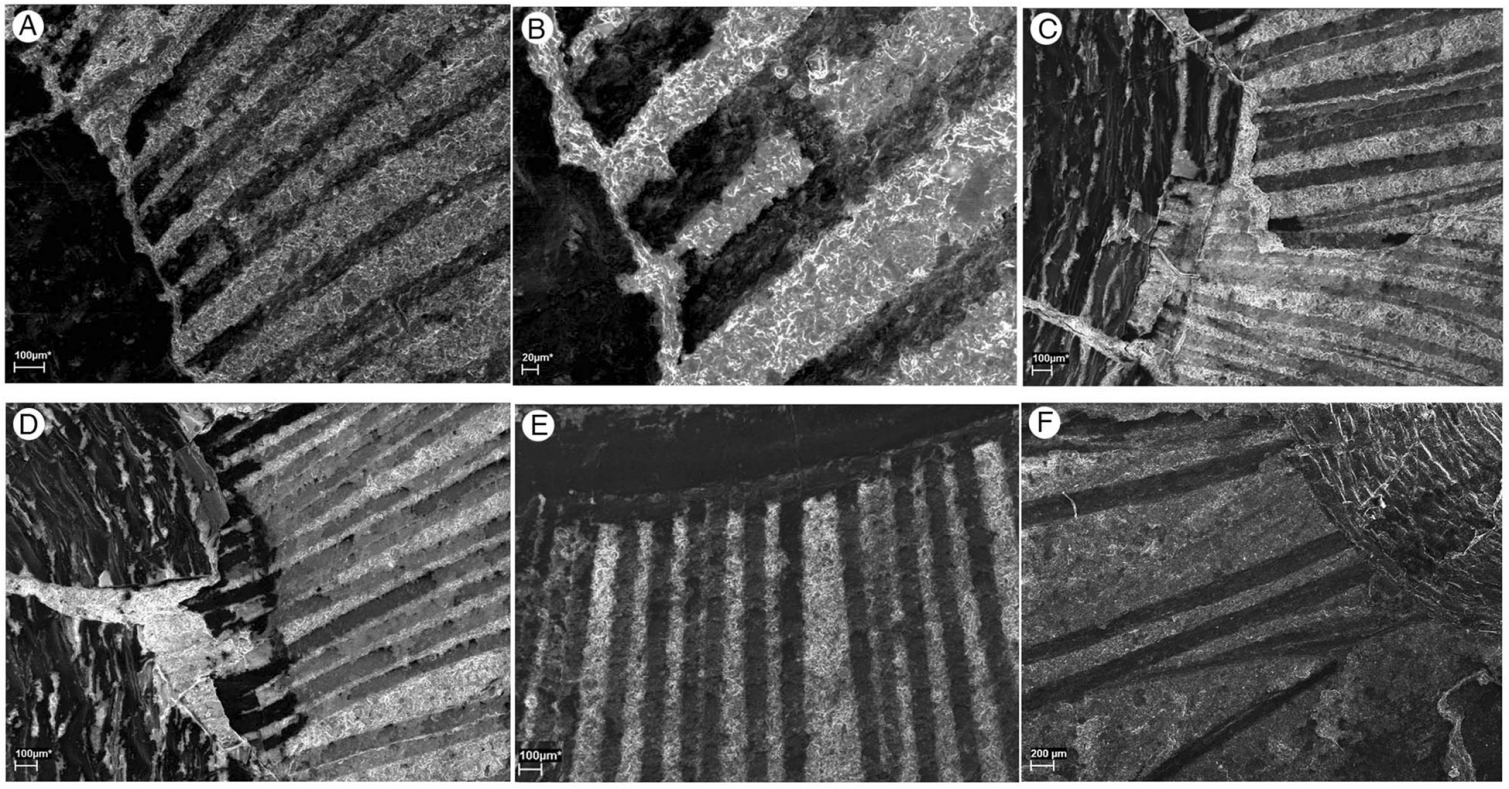
**Scanning Electron Microscope images of brachiopods from the middle (Series 3, Stage 5) Cambrian Burgess Shale. A-B**, ROM63169, RQ + 8.2 m, anteromedial chaetae of *Micromitra burgessensis*, **B**, scale bar 20 μm. **C-D**, ROM57603, BW-120 cm, anteromedial chaetae of *Micromitra burgessensis*. **E**, ROM63185, Talus above Walcott Quarry, anteromedial chaetae of *Paterina zenobia*. **F**, GSC 81224, Walcott Quarry, Phyllopod Bed, *Pirania muricata* spicules emerging from underneath *Micromitra burgessensis*, scale bar 200 μm. Scale bars 100 μm unless otherwise stated.

**Figure 4:**
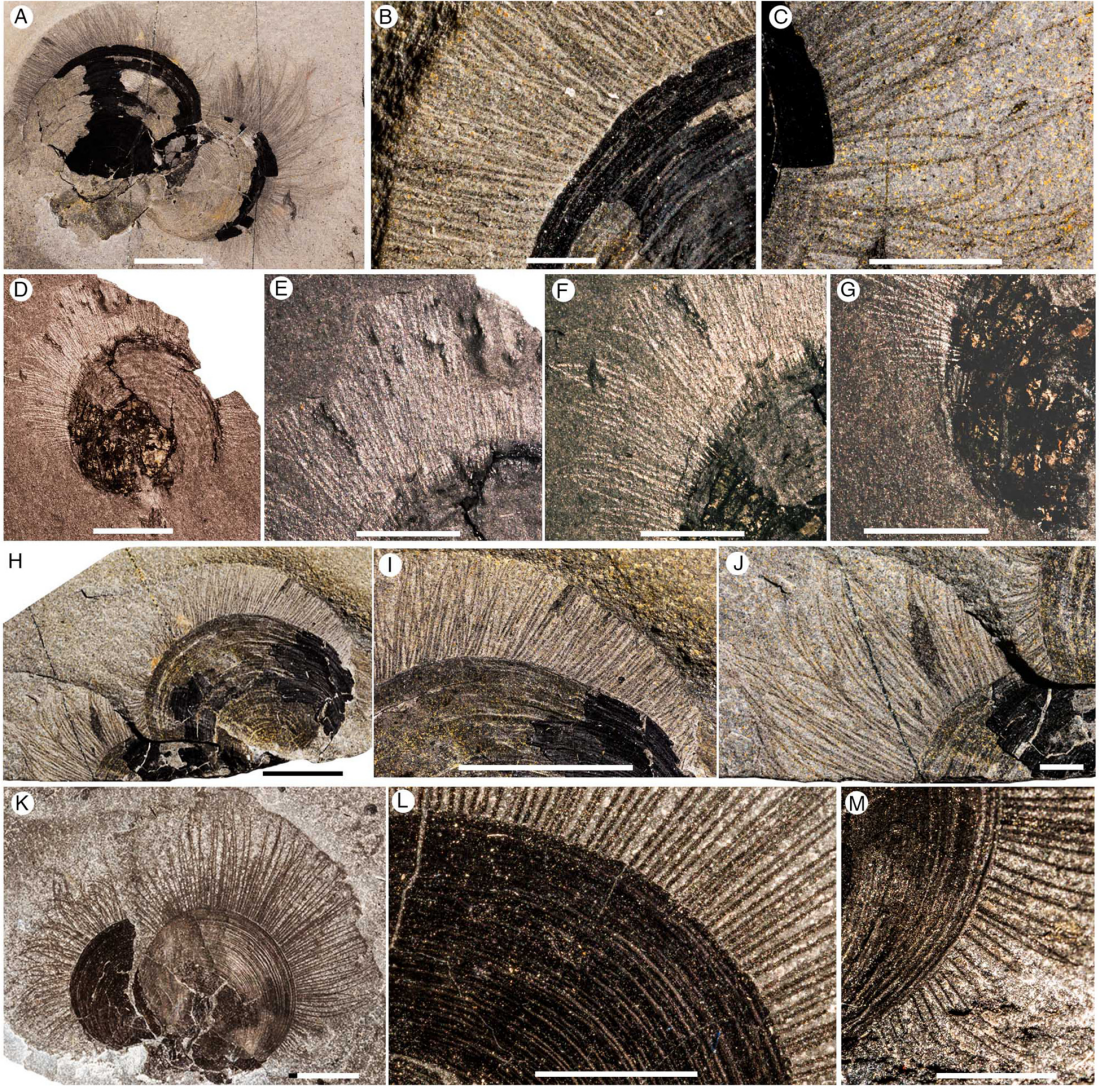
***Paterina zenobia*** **from the middle (Series 3, Stage 5) Cambrian Burgess Shale. A-C**, ROM63182, WT talus. **A**, Plan view of specimens attached to a trilobite carapace, scale bar 5 mm **B**, Close up of anteromedial chaetae, scale bar 1 mm. **C**, Close up of posterolateral chaetae, scale bar 1 mm. **D-G**, ROM63183, S7; Tulip Beds Talus (Mount Stephen). **D**, Plan view, scale bar 5 mm. **E**, Close up of anteromedial chaetae. **F**, Close up of posterolateral chaetae. **G**, Close up of posterolateral chaetae. **H-J**, ROM63184, WT talus. **H**, Plan view, scale bar 5 mm. **I**, Close up of anteromedial chaetae, scale bar 5 mm. **J**, Close up of posterolateral chaetae, scale bar 1 mm. **K-M**, ROM63185, Talus above Walcott Quarry. **K**, Plan view, scale bar 5 mm. **L**, Close up of anteromedial chaetae. **M**, Close up of posterolateral chaetae. Scale bars 2.5 mm unless otherwise stated.

Walcott’s [39,40] documentation of *Micromitra* from the Burgess Shale Lagerstätte in western Canada represents the first report of brachiopod chaetae in the fossil record. Arguably the most significant and influential of the Cambrian Lagerstätten, the brachiopods from the Burgess Shale have seen little attention since these original descriptions [5,6,39-42]. Here, we examine one of the more remarkably preserved brachiopods from the middle (Series 3, Stage 5) Cambrian Burgess Shale, *Micromitra burgessensis* Resser [41]. *Micromitra* is exceptionally preserved, exhibiting elongate chaetae that fringe the mantle and extend far beyond the margin of the biomineralized shell (Figures 1, 2 and 3A-E). The shape, size and frequency of the chaetae possessed by *M. burgessensis* are unique amongst extant and fossil brachiopod taxa and herein we provide explanations for their functional significance based upon comparisons with extant brachiopod chaetae and chaetae of other lophotrochozan groups. Specimens of a second brachiopod taxon, *Paterina zenobia* Walcott, [40] are also discussed, as a number of specimens exist with preserved chaetae (Figures 3E and 4). The function of chaetae in fossil brachiopod taxa is perhaps even more difficult to assess than for extant forms, yet as chaetae represent such a distinctive morphological feature for both *M. burgessensis* and *P. zenobia*, their potential function demand attention. Both *Micromitra* and *Paterina* are globally distributed genera across Cambrian Series 2–3 [43,44] and their taxonomic position in one of the oldest brachiopod families (the Paterinata) provides important clues about the evolution, ecological and phylogenetic significance of lophotrochozoan chaetae.

## Results

### *Micromitra burgessensis*

*Micromitra burgessensis* can be readily identified in the Burgess Shale Formation from its distinctive diamond-shape ornament (Figures 1 and 2). The valves of *M. burgessensis* reach a maximum width of 12.1 mm and a maximum length of 10 mm. Specimens (58 individuals) are commonly found attached to a variety of substrates, most commonly (32 out of 58 individuals) on the spicules of the sponge *Pirania* (Figures 1F-J and 2J-M) but also on *Tubulella* (Figure 1A-E) and on other individuals of *M. burgessensis* (Figures 1I and 2C). Of the 208 specimens of *M. burgessensis* examined from the Burgess Shale Formation, a total of 101 (48%) individuals were preserved exhibiting identifiable chaetae. The chaetae fringe the dorsal and ventral valve margins and are generally at their greatest length anteromedially, decreasing in length posterolaterally as they approach the hinge line, where they disappear entirely (Figures 1 and 2). The individual chaeta are elongate and taper to a pointed terminus. The chaetae are evenly distributed across the margin of the shell giving the brachiopods a rough bilateral symmetrical appearance. Commonly curved, but never twisted, the chaetae radiate out from the valve margins and probably possessed a degree of flexibility in life. Evidence from broken chaetae (Figure 2D) however, suggests that the chitinous structures, despite their flexibility, possessed some degree of rigidity and brittleness. The presence of dissociated valves that have preserved chaetae suggests the chaetae decay at a slower rate than the muscles that hold the two valves together (Figure 2I). The chaetae can be seen emerging from underneath the shell in specimens where the shell margins are broken (Figures 1Q, 2B and 3D), however no additional internal soft tissue (e.g. mantle, lophophore) has been observed.

The total number of chaetae and spacing between each chaeta as they emerge from the valve margin of individuals is variable and difficult to measure accurately since the chaetae associated with one or both of the valves may be variably preserved (compare Figures 1K-M and 2N-R with Figures 2A-B). Specimens that possess chaetae that are spaced at approximately 60–80 μm intervals, with a maximum number of 84 chaetae, are interpreted as representing specimens where chaetae associated with only one valve is preserved. Specimens that possess chaetae that are spaced at approximately 20–50 μm intervals, with a maximum total number of 144 chaetae, are interpreted as representing specimens where the chaetae associated with both ventral and dorsal valves are preserved on the same surface. The chaetae of *M. burgessensis*, in respect to shell size are remarkably long (maximum length 11.7 mm). On average, the maximum chaetal length is the same length of the shell from which it projects (102% of shell length, n = 30) but can in rare circumstances exceed twice the maximum length of the shell. The posterolateral chaetae are usually shorter than their anteriomedial counterparts and typically measure close to half the width of the brachiopod shell (maximum length 6 mm). With both posterolateral sides taken into account, this can double the width of the entire brachiopod specimen. Individual chaeta vary in width from 67 to 100 μm (mean 81 μm, n = 50) and show a tendency to become thinner on the posterolateral sides of the valve. A small individual, interpreted as representing a juvenile (width of 2 mm, length of 1.75 mm, Figure 1O) also possesses chaetae. The chaeta in this case are 1.5 mm in length (85% of the shell length) and fewer in number (total number of chaetae 36). No chaetal ultrastructure is obvious from the specimens examined.

### *Paterina zenobia*

*Paterina zenobia* broadly resembles *Micromitra burgessensis* in general shell morphology and was originally assigned to *Micromitra* [40], however the differences in ornament (prominent growth lines versus diamond shaped ornament) differentiate the two taxa. *Paterina zenobia* is only known from talus material and despite being found in material overlying the Walcott Quarry (presumably derived from the younger Raymond Quarry Member), the species has yet to be identified *in situ*. Seventy four specimens of *P. zenobia* were examined, of which 19 (26%) were preserved exhibiting identifiable chaetae. The valves of *P. zenobia* are slightly larger than *M. burgessensis*, reaching a maximum of 15 mm in width and 14 mm in length and like *M. burgessensis* are found attached to a variety of substrates, such as trilobite carapaces (Figure 4A-C) and other individuals of *P. zenobia*. The chaetae in the majority of these specimens were not complete and measurements and counts were possible for only five individuals (Figure 4). One specimen of *P. zenobia* is preserved attached to *Pirania*, however no identifiable chaetae are apparent.

The chaetae of *P. zenobia* bear many similarities to those of *M. burgessensis*, fringing the valve margins and are at their greatest length anteromedially, decreasing in length posterolaterally as they approach the hinge line, where once again they disappear entirely (Figure 4). The chaetae are elongate and taper to a point and their curved and swept nature also suggests a degree of flexibility in life (Figure 4A). The chaetae are not as long as observed in *M. burgessensis*, reaching a maximum length of 7.1 mm (67% of shell length), and on average (n = 5) only extend approximately 55% of the entire shell length (Figure 4). The posterolateral chaetae also extend the width of the individual by up to 7 mm (3.5 mm on each posterolateral side), an increase in the width of the individual of 70% (Figure 4A). Individual chaeta varies in width from 77 to 114 μm (mean 99 μm, n = 50) and decrease slightly in width on the posterolateral sides of the valve. The total number of chaetae for each individual is problematic as parts of the shell on many specimens (Figure 4) are obscured or damaged, leaving an incomplete margin. For all chaetae-bearing individuals it is interpreted that the chaetae from both the ventral and dorsal valves are visible. The most complete specimen (ROM63182; Figure 4A) possesses approximately 175 chaetae, spaced at intervals of 20–40 μm. The remaining individuals exhibited a range of 122 to 146 chaetae, spaced at similar 20–40 μm intervals, however it is assumed that a number of chaetae are not preserved, since the shell margin is incomplete (Figure 4). No juveniles of *P. zenobia* possessing chaetae have been observed.

## Discussion

### Functions and mechanisms of chaetae

**Figure 5:**
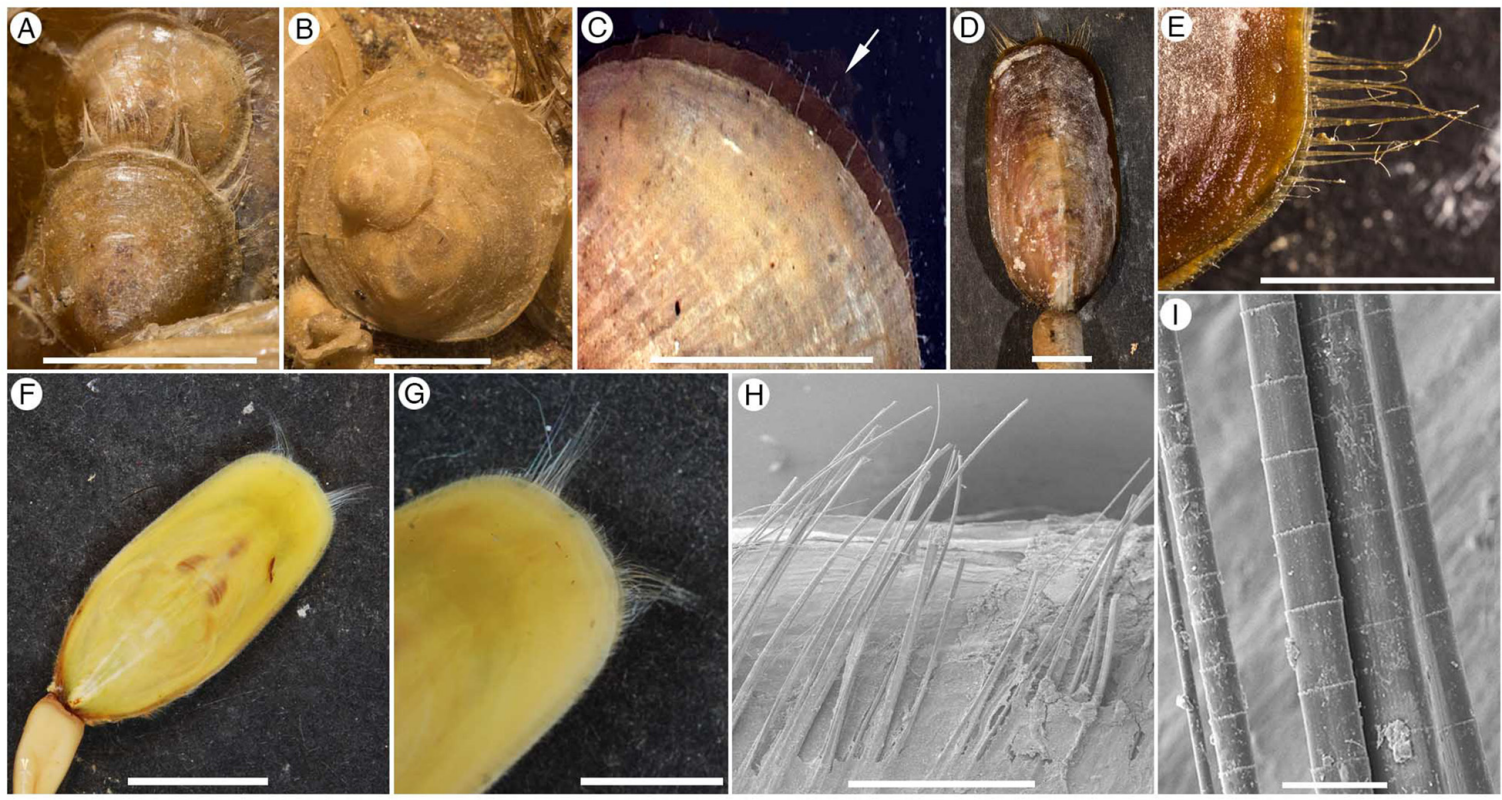
**Extant brachiopod genera. A-B**, *Discinisca lamellosa*, Namibia. **A**, SMNH141416, Gregarious cluster of individuals exhibiting chaetae. **B**, SMNH141417, Gregarious cluster of individuals exhibiting chaetae.**C**, *Terebratulina retusa*, west coast of Sweden, Arrow directed towards marginal chaetae. **D-E**, *Lingula adamsi,* SMNH141418, Beidaine, Hebei Province, China. **D**, Plan view, scale bar 1 cm. **E**, Close up of anterolateral chaetae. **F-I**, *Lingula anatina*, **F-G**, SMNH 140566, Ariake Bay, Japan. **F**, Plan view, scale bar 1 cm. **G**, close up of anterior chaetae. **H-I**, SMNH141419, Cebu Island, Philippines. **H**, Anterior chaetae, scale bar 1 mm. **I**, Magnification of chaetae, scale bar 50 μm. Scale bars 5 mm unless otherwise stated.

The chaetae of *Micromitra burgessensis* and *Paterina zenobia* contrast significantly with those of extant brachiopods. For example, the average diameter of *M. burgessensis* and *P. zenobia* chaetae are 81 μm and 99 μm respectively, compared with the chaetae of extant taxa, such as *Discinisca strigata* (Figure 5A-B), that measure 13 μm in diameter [33] and the chaetae of *Lingula anatina* that are approximately 14 μm in diameter (Lüter [19], Figure one). This is despite the comparable shell size of all these taxa. The chaetae possessed by *M. burgessensis* and *P. zenobia* also reach a greater length relative to shell size when compared to the majority of extant taxa. Only the chaetae of discinids are comparable (Figure 5A-B), extending approximately 80% of their maximum shell length [33,45]. Given their size and prominence, it is certain that the chaetae of both *M. burgessensis* and *P. zenobia* have important functional significance. Their construction would represent a significant investment to the organism. They also represent a potential liability, extending well beyond the shell margin, their size potentially inhibiting the tight closure of the valves and thus increasing the vulnerability of the individual to predators. Based upon a comprehensive review of the Burgess Shale fauna, both fossil and extant brachiopods and similar structures in other organisms, we herein propose and provide evidence for and against a number of potential functions for chaetae in *M. burgessensis* and *P. zenobia* These functions are: sensory and defence; feeding and defouling; mimicry and spatial competition.

### Sensory function

Brachiopod chaetae are frequently interpreted as sensory grilles, extending the tactile sensitivity of the mantle beyond the margin of the valves by projecting their chitinous chaetae into the environment [21,25,32,46]. Despite this generally accepted sensory role for brachiopod chaetae, it has never been critically evaluated and very little is known about how chaetae function as sensory features in living brachiopods [25]. A response to some form of tactile stimuli is one option, the long projections acting as an early warning system when stimulated by potential predators. Living brachiopods most certainly respond to tactile stimuli, but the chaetae do not appear to have any direct connection to the nervous system [25] and no specialized sensory chaetal cells have been identified [25,32]. The mantle edges of living brachiopods are richly supplied with nerves, and any tactile stimulus or chemical imbalance at the mantle edge causes an immediate and rapid closure of the shell [32,46,47]. This appears to be the primary protective reaction in living brachiopods, the closure of the shell protecting the soft-tissue from potentially harmful agents in the external environment [32]. There currently exists no published evidence that protective sealing of the shell is directly linked to the stimulation of the brachiopod chaetae, only to the mantle edge itself. As projections of the mantle, movement of the chaetae presumably stimulate sensory organs within the mantle, however no research in extant taxa, exists to confirm this.

Whilst the sensory effectiveness of brachiopod chaetae is questionable, it is possible the long, closely-spaced, somewhat rigid chaetae of *Micromitra burgessensis* and *Paterina zenobia* may have constituted a protective grille, inhibiting access to the mantle tissues. The unusual length and thickness (compared to Modern forms, see Figure 5) and high density of chaetae would severely impede the potential predator, regardless of whether the valves could shut completely. The potential effectiveness of this protective grille may explain the lack of durophagy on the shell edge of *M. burgessensis* and *P. zenobia* individuals, as documented in other Cambrian deposits [48-50]. Brachiopods have been documented in the gut of *Ottoia prolifica* [51-53] a priapulid worm, and the large arthropod, *Sidneyia inexpectans* [52,54] and possibly the only effective method of predation on brachiopods was the entire digestion of the brachiopod individual as seen in *Ottoia* [51] or the grasping and crushing of the individual into ingestible fragments as seen in *Sidneyia* [52]. For both these modes of feeding, any early warning system provided by the chaetae, and the associated closure of the valves, may have been an inadequate protective reaction. Another possibility is that the substantial increase in body size of chaetae-bearing brachiopod individuals was a deterrent against predators. The illustrated *M. burgessensis* individuals in the gut of *Ottoia* [51] are all relatively small (approximately 2–3 mm in width) and the possession of long, semi-rigid chaetae may have made the task of ingesting the larger brachiopod prey mechanically difficult for *Ottoia*. The fragmented nature of *M. burgessensis* valves in the gut of *Sidneyia* [52] prevents a corresponding measurement.

Brachiopods were certainly prey items in the Burgess Shale community however the low percentage of brachiopod individuals in examined gut contents and coprolites [51,52] may reflect the effectiveness of the chaetae as sensory tools. The exaggerated length of the chaetae in *M. burgessensis* and *P. zenobia* would have potentially provided an earlier warning system in comparison to much shorter marginal chaetae (Figure 5C), allowing sufficient time to close their shells and increasing their chance of survival. Direct evidence of durophagous predation, whether represented by boreholes, drillholes or shell-breaking bite marks is rare in brachiopod specimens from the Burgess Shale. A similar story is reported from the Chengjiang Lagerstätte, where there are no recognized examples of durophagous activities [48] and brachiopods are also conspicuously absent from early Cambrian coprolite aggregates [49]. Repaired durophagus shell damage has been recorded in brachiopods from the Cambrian Wulongqing Formation (Series 2, Stage 4) of China, although only thirteen specimens of *Diandongia pista* [55] out of over 1400 specimens in total are documented with predatory shell damages [48]. This apparent lack of predation on individuals suggests that, brachiopods possessed excellent defensive mechanisms against predators.

Ideally, any effective defense system would see the sensitive mantle edges projected not only outwards, but also dorsally and ventrally from the apertures, covering the entire shell margin to protect from harmful agents approaching the individual from every direction. It would be difficult to reconcile this morphological specification with the structural requirement for protection, which is the tight sealing of the valves. Additionally, the large majority of extant brachiopods, such as the lingulids [21] can retract their chaetae back into their shell, allowing their shells to securely close, protecting the soft tissue. The sheer length, number of chaetae present and their semi-rigidity suggests that, for *M. burgessensis* and *P. zenobia,* chaetal retraction would not be possible, resulting in incomplete closure of the valves and potentially weakening their primary defensive system. The large majority of other shell bearing lophotrochozoans, such as bivalves and gastropods, possess the ability to fully enclose their soft parts within their protective shell [56]. Individuals with an open gape would likely be much more susceptible to predation, with likely lower fitness compared to individuals that could tightly close their shell. Consequently such individuals are rare in modern marine communities.

The possibility also exists that the low percentage of brachiopods in predator gut contents may indicate that brachiopods were rarely chosen as prey. Many authors consider brachiopods, both in the current marine realm and in the geological past, as not having been significant prey items [32,57-61]. This is predominantly due to the lack of detailed studies on living and fossil taxa and laboratory observations that show a variety of modern predators (e.g. fish, gastropods and asteroids) actively preferring bivalve mussels over brachiopods [57] or avoiding brachiopod individuals altogether [58]. Nevertheless, there are records of predation on Recent brachiopods [62,63] and despite the tenuous evidence from Cambrian Lagerstätte deposits, there are have been intermittent accounts of predation on brachiopods throughout the Palaeozoic [50,61,64,65]. Brachiopods were faced with predation pressure during the Cambrian and certainly in the Burgess Shale, however with the exception of *Ottoia* and *Sidneyia*, direct evidence of other brachiopod predators in the Burgess Shale is meager. It is also difficult to rationalize, that if the unique length of *M. burgessensis* and *P. zenobia* chaetae evolved as effective sensory tools against predation, why haven’t similar sized structures been employed in a similar manner by subsequent brachiopod lineages?

### Feeding and modifying flow

Activity in sessile, attached brachiopods is essentially restricted to the opening and closing of the valves and the near-continual beating of the lateral cilia of the lophophore, allowing the organism to feed by bringing in various nutritional sources from the water [32,66]. Living brachiopods have been observed to be quite selective in the capturing of particles for ingestion and have the ability to reject unwanted excess mud and silt from the lophophore and mantle cavity by reversing the direction of the lophophore ciliary beat [67-70]. Spines and chaetae have previously been suggested as acting as a feeding sieve, forming interlocking grilles around the commissural gape, ensuring effective sieving of inhalant currents and protection against larger less desirable particles [32,37]. Protection against larger particles would not have been necessary for the benthic Burgess Shale community that predominantly lived on a homogeneous fine-grained mud seafloor [71,72]. A fine-grained muddy seafloor would, however, have increased the likelihood of ‘clogging’ the brachiopod lophophore with the accumulation of suspended fine particles in the mantle cavity, leading to an inability to either intake food or respire or both, and ultimately leading to the death of the organism. The possibility therefore exists that the chaetae of *Micromitra burgessensis* and *Paterina zenobia* represent a mechanism to obstruct the excessive intake of fine particles.

Chaetae in specimens of *M. burgessensis* are spaced at approximately 20–50 μm and 20–40 μm in *P. zenobia*. This measurement was taken at the anterior shell margin, and is interpreted as representing the chaetae from both dorsal and ventral valves, signifying that the valves are preserved closed. Space between the chaetae would have increased upon the opening of the valves. With petrographic analyses indicating that particles in the Burgess Shale claystones were originally probably less than 25 μm [72], the chaetae of *M. burgessensis* and *P. zenobia* would not have acted as an effective screening tool for limiting the amount of fine particulate matter entering the mantle and the clogging of the lophophore. The open gape of the extant genera, *Terebratalia* and *Liothyrella* is frequently held at the maximum angle that can be covered by the projecting chaetae, an action that has been used to support the interpretation of the chaetae acting as a grill or strainer to keep out sediment and other large particles from the mantle cavity [32,69]. The chaetae of *Terebratalia* and *Liothyrella* however are short relative to shell size, approximately 20% of shell length [69,73]. Such an action would not be physically possible in *M. burgessensis* or *P. zenobia,* as this would require the individual to have an open gape twice the length of its valves. The lophophore of a paterinid brachiopod has never been documented, however previous authors have suggested the removal of unwanted fine particles from the mantle cavity of extant brachiopods is likely to be reliant on the lophophore [69] and not dependent on chaetae.

Some extant brachiopods utilize their chaetae to form siphons to either assist in feeding [33,45] or burrowing into the substrate [21,38]. For example, the anterior chaetae of *Discinisca strigata* [74] bear fine lateral processes which mechanically interlock forming an incurrent siphon that extends above the substratum and other nearby epifauna, allowing for an enhanced current and improvement in feeding [33,45]. With both species of paterinid Burgess Shale brachiopod species documented attached to a variety of substrates, utilizing their chaetae to assist in a burrowing process can be discounted. Neither *M. burgessensis* nor *P. zenobia* show a comparable chaetal clustering to *Discinisca* (Figure 5A-B) and, consequently, it is unlikely that their chaetae were used to form a siphon. As many *M. burgessensis* specimens are attached to the sponge *Pirania muricata* in the Burgess Shale ecosystem (56% of attached *M. burgessensis* specimens), a siphon to enhance current for feeding purposes may not have been necessary, as *M. burgessensis* individuals could have taken advantage of nutrients, through currents, however small, produced by the sponge [75,76].

### Mimicry

Mimicry is commonly thought to involve the resemblance of one organism (described as the mimic) to another organism (described as the model), the physical similarity adopted by the mimic, a mechanism to deceive a third organism [77-79]. Harmless and palatable organisms frequently mimic, dangerous and/or unpalatable organisms to avoid interest from potential predators, a form known as Batesian mimicry [78-82]. Mimicry has been interpreted across a range of marine lophotrochozoa taxa, including polychaetes [78], chaetognaths [78] and a variety of molluscs [79,83]. Mimicry, as a concept, has rarely been applied to brachiopods and many of the previous examples possibly represent homeomorphy or camouflage rather than mimicry [84]. A major hurdle in understanding mimicry is that it is notoriously hard to detect, even in extant faunas, as evidence for mimicry is predominantly a result of direct observation of the organisms in question [77,85-88].

**Figure 6:**
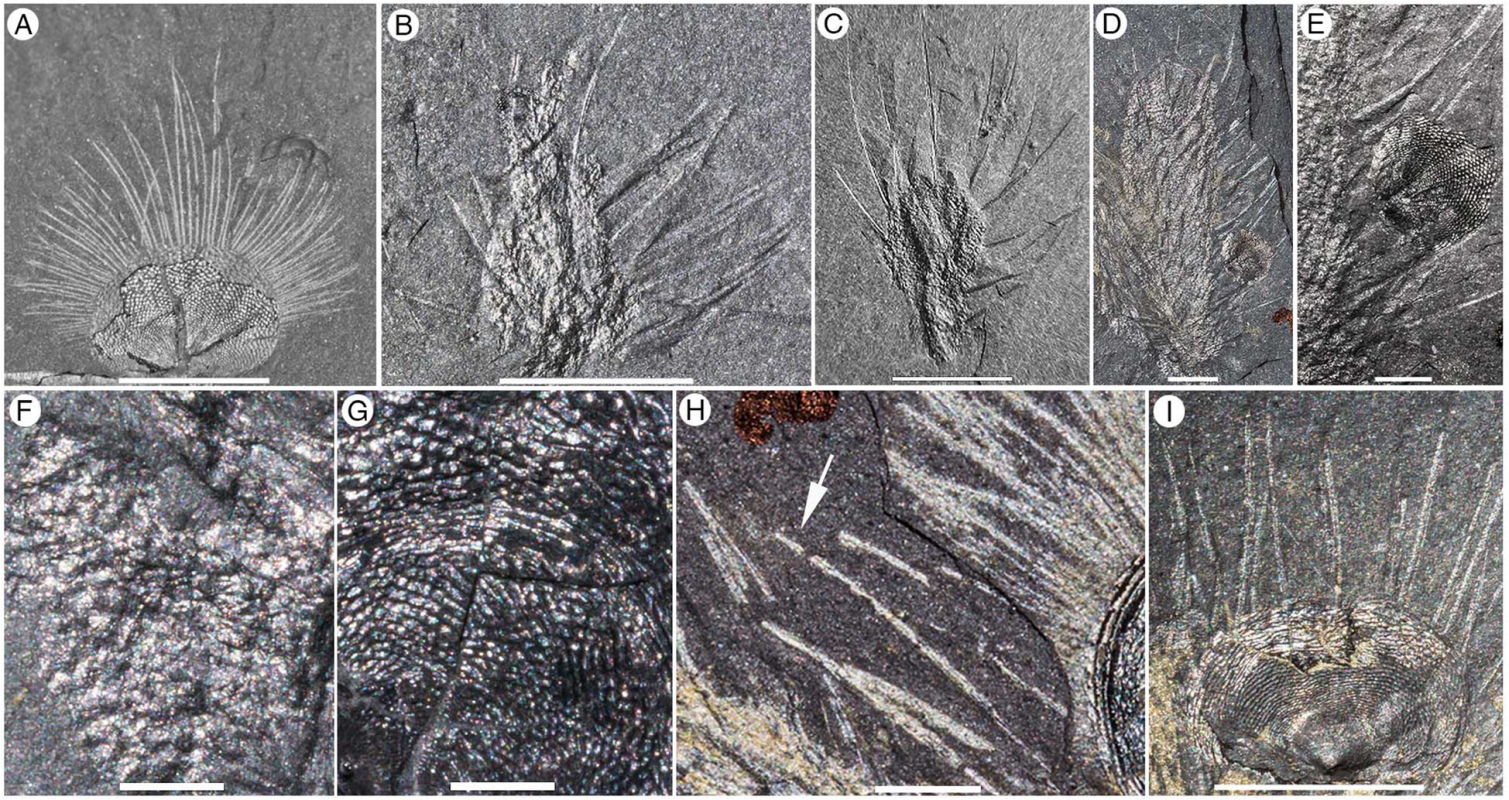
***Micromitra burgessensis*** **and** ***Pirania muricata*** **from the middle (Series 3, Stage 5) Cambrian Burgess Shale. A,** ROM63169, RQ + 8.2 m, plan view of *M. burgessensis*. **B**, ROM63186, BW-210 cm, Plan view of *P. muricata*. **C**, ROM63188, BW-210 cm, Plan view of *P. muricata*. **D-E**, ROM63189, BW-170 cm. **D**, Plan view of *P. muricata* and *M. burgessensis*, scale bar 2 mm. **E**, close up of *M. burgessensis* and the sponge wall of *P. muricata*, scale bar 1 mm. **F-G**, ROM61135, BW-200 cm. **F**, Close up of rhomboidal texture of the sponge wall of *P. muricata*, scale bar 1 mm. **G**, Close up of shell ornament on *M. burgessensis*, scale bar 1 mm. **H**, ROM63187, WQ-170 cm, Close up of *P. muricata* spicules (arrow is used to indicate sponge spicules on the left of the image) juxtaposed with *M. burgessensis* chaetae (right of the image), scale bar 1 mm. **I**, GSC 81224, Walcott Quarry, Phyllopod Bed, Plan view of *M. burgessensis* with *P. muricata* spicules emerging from underneath the shell. Scale bars 5 mm unless otherwise stated.

*Micromitra burgessensis* and *Paterina zenobia* are preserved in the Burgess Shale Formation attached to a variety of substrates, including the demosponge *Vauxia*, the enigmatic *Chancelloria*, a variety of disarticulated skeletal elements and conspecific individuals (Additional file 1). The majority of chaetae-bearing *M. burgessensis* specimens (56% of attached individuals) are preserved perched on the cactus-like sponge, *Pirania muricata* [89]. *Pirania* is characterized by distinctive coarse, long monaxial spicules [90], that radiate upward and outward from the branch of the moderately thick-walled sponge (Figure 6B-D). It is these long spicules to which *Micromitra* individuals commonly attach to (Figures 1G, 2K and 6D-E). *Paterina zenobia* specimens are preserved attached to *Pirania muricata* (12.5% of attached individuals), however the lack of *in situ* material, limits further detailed ecological comparisons and studies.

*Pirania muricata* is interpreted as representing an unpalatable organism to the majority of predators, their siliceous skeleton offering low nutritional value, analogous to modern siliceous sponges [91]. The lack of predation marks on *Pirania* specimens and the lack of *Pirania* spicules in the contents of guts or coprolites [49,51] support this interpretation. Spongivory in modern communities is largely restricted to a few species of fish [92], nudibranchs [93] and turtles [94,95] all absent from Cambrian faunas. *Pirania* though, may not be totally immune from predators, with claims that lobopodians, such as *Aysheaia* and *Hallucigenia* fed on sponges in the Burgess Shale community [96]. This suggestion is largely hypothetical and based entirely on ecological associations [96]. Recent investigation of gut contents in lobopodians from Chengjiang and the Burgess Shale Lagerstätten [97] provided no evidence to support this theory and sponge spicules have not been reported from the gut contents of either *Ottoia* or *Sidneyia* [51,52]. It is conceivable that the long, monaxial spicules possessed by *Pirania* are defensive structures, offering protection from potential predators. Similar structures however, have also been interpreted as offering body support for erect sponges [98]. There is currently no direct evidence to suggest high rates of predation in *Pirania* and brachiopods were likely more frequently predated upon and seen as a more palatable prey item in the Burgess Shale ecosystem.

The individual radiating spicules of *Pirania* typically measure 7–8 mm in length, are approximately 100–200 μm in width (Figure 3F) at the base of the spicule and taper to a point [90]. These dimensions are very similar to the length and width of chaeta possessed by *M. burgessensis*. The similarities are further apparent in samples where *M. burgessensis* valves are preserved overlaying *Pirania* spicules (Figure 6I), where the spicules could easily be mistaken for the chaetae of *M. burgessensis. Micromitra burgessensis* individuals with a full chaetal complement reach a maximum size of 23 mm in length and 26 mm in width. Although *Pirania* varies in size, minimum 6.5 mm in height and 4 mm in width (Figure 1F) and maximum 30 mm in height and 13 mm in width [90], the size of *M. burgessensis* and *Pirania* individuals are largely comparable. There is also a remarkable similarity in the diamond-shaped shell structure of *M. burgessensis* (though absent in *Paterina zenobia*) and the rhomboidal texture of the tufts and canals of the sponge wall of *Pirania* (compare Figure 6F and G).

The prevalence of *Micromitra burgessensis* individuals attached to *Pirania* (Additional file 1) suggests the two organisms frequently lived in the same ecosystem. Previous analyses of the Burgess Shale community have also demonstrated a close association between the two organisms and that this interaction was constant over time [99]. Consequently, potential brachiopod predators (e.g. *Ottoia* or *Sidneyia*) in the Burgess Shale fauna must have also encountered *Pirania*. The chaetae exhibited by *M. burgessensis* and *P. zenobia* have not been replicated by other fossil or extant brachiopods and long, semi-rigid chaetae are seemingly restricted to the Burgess Shale, where the sponge *Pirania* is most common. Combined with the close faunal associations of *M. burgessensis* and *Pirania* and the size similarities between the spicules of *Pirania* and the chaetae of *M. burgessensis,* this raises the possibility that *M. burgessensis* chaetae evolved to mimic characteristics of a co-occurring unpalatable sponge and deter potential predators. This relationship would constitute a form of Batesian mimicry, where palatable species mimic an unpalatable model, obtaining some degree of protection by deceiving predators that have learnt to avoid the unpalatable model [77,85,86]. For Batesian mimicry to successfully result in lower mortality rates it is crucial that the unprofitable model is present in the community at high frequency [77,85,86]. A greater proportion of models compared with mimics is observed in the Burgess Shale community where *Pirania* individuals outnumber *M. burgessensis* individuals (253 specimens of *Pirania* compared to 134 specimens of *Micromitra* in the Walcott Quarry Member) [99,100]. If the model is absent from the community, there is potential that predators will not recognize the mimic as unpalatable and the protection provided by the model could collapse [88]. *Pirania* is absent from the Burgess Shale community documented from the Raymond Quarry (slightly younger than the Walcott Quarry – [90]). Specimens of *Ottoia* that contain *Micromitra* valves in their gut are all part of the Raymond Quarry community, with only one possible example of a small *M. burgessensis* specimen in the gut of an *Ottoia* from the older Walcott Quarry, where *Pirania* is present (Vannier [51] Additional file 1: Table S1). While this disparity may be due to variable taphonomic biases that influence abundance levels [100], that this congruence with modern studies exists [88] does further support the possibility that Batesian mimicry explains the uniqueness of the chaetae exhibited by *Micromitra burgessensis*.

*Micromitra burgessensis* and *Pirania muricata* may not be morphologically identical, however examples of the imperfect resemblance between Batesian mimics and their models are widespread [101-103]. For example, many species of hoverflies are generally regarded as Batesian mimics of wasps and bees and yet to the human eye, the resemblance between the organisms is quite crude [101,103,104]. Batesian mimicry is somewhat dependent on the visual capabilities of the predators and mimics will resemble their models in ways that potential predators can perceive [105,106]. The morphology of mimics may simply evolve to the extent where the resemblance to their model is sufficient enough to deceive the visual capabilities of the predator [105-107], which may be the case for *M. burgessensis*.

### Spatial competition

One of the most important resources a brachiopod must compete for is space, a necessary requirement for successful settlement, growth and feeding [25,108]. Substrate space is frequently a limiting resource and, as an immobile component of the hard substratum epifauna, brachiopods have been competing for living space with other epifaunal organisms since the Cambrian. Encrusting sponges and colonial bryozoans are generally considered in modern marine communities to be superior competitors when competing with solitary animals for substrate space [109-111] and the limited passive defenses of living brachiopods make them particularly vulnerable to smothering by other organisms [25,111].

Brachiopods typically struggle to discourage competitors’ growth, relying upon their large size, coupled with frequent shell rotation around the pedicle and raising the commissure of their shell off the substrate to impede growth of spatial competitors [112-114]. Brachiopod species tend to be gregarious in shallow water habitats where spatial competition is assumed to be intense and this lifestyle may represent another potential mechanism to exclude competitors, grazers and predators [33,62,114-116]. Brachiopod larvae are commonly documented settling on conspecifics, frequently on the anterior edge of the maternal shell (Figure 5B), leading to the formation of dense ‘grape-like’ clumps of brachiopod individuals [45,57,108,113,117]. This strategy is evident in Burgess Shale specimens (Figures 1I and 2C). In laboratory conditions, the larvae of *Laques* and *Liothyrella* preferentially settle on the shells of living conspecifics [108,117] and in some cases conspecific shells even induce the metamorphosis of the larvae brachiopod [115,118]. The stunting and malformation of some individuals growing within these clumps [57,113] are indications that some negative effects of conspecific settlement do exist. However potential advantages of reduced juvenile mortality and excluding competitors and grazers must outweigh these negative effects, given the frequency of these gregarious clumps [108,117]. One impressive account of active defense against spatial competitors is the discinid brachiopod *Discinisca strigata*, that prevents or represses the growth of competitive invertebrates by abrading their tissues and depleting the immediately surrounding water of nutrients [33]. The abrasion is generated by the brachiopod closing and rotating its phosphatic valves, resulting in a sweeping motion of its long, robust barbed chaetae (Labarbera [33], Figure three) damaging the tissues of the adjacent bryozoans and encrusting sponges [33].

The soft, muddy Burgess Shale seafloor would have provided limited hard substrates for brachiopods to attach to, including largely biogenic substrates, such as sponges (Figures 1F-J and 2K-M), other brachiopods (Figure 2C) and disarticulated skeletal elements (Figures 1K and 4A). This need to settle on a hard substrates with only limited suitable substrates available presumably resulted in spatial competition among the dominant sessile members of the epifaunal Burgess Shale community. The large majority of attached specimens (Additional file 1) of *Micromitra burgessensis* and *Paterina zenobia* display a solitary lifestyle (the only organism attached to that particular substrate) or are conspecific clusters of individuals (Figure 1E, I). There are only three cases (Figures 1F-H, J) where *M. burgessensis* individuals share a substratum (*Pirania muricata*) with another taxon; one with the acrotretid *Acrothyra gregaria* (Figure 1F), one with the kutorginid *Nisusia burgessensis* (Figure 1G-H) and one with both an *A. gregaria* and *N. burgessensis* (Figure 1J). In all three cases, the brachiopod individuals are positioned on opposite sides of *Pirania* with the anterior edge of the shell oriented away from the other brachiopod individuals (Figure 1F-J). This positioning suggests that no *M. burgessensis* individuals on the specimens examined would have had the opening of their valves impeded by the presence of *A. gregaria* or *N. burgessensis*, with appropriate space between the individuals (Figure 4G-H).

In terms of chaetal length relative to shell size, *M. burgessensis* and *P. zenobia* are most comparable to extant discinids, in particular *Discinisca* (Figure 5A-B) and *Pelagodiscus* [119]. Both discinid taxa possess chaetae that commonly exceed the length of the shell by a few millimeters [33,45,119]. The musculature of both *M. burgessensis* and *P. zenobia* would not have allowed for a discinid-like rotation of the valves [43,44] and there is no evidence of barbs on the chaetae of either paterinate taxa. However, the brachiopods of the Burgess Shale did not have to spatially contend with encrusting sponges and bryozoans [99]. The main competition for attachment space on hard substrata would have been from other brachiopod individuals. The chaetae of *M. burgessensis* and *P. zenobia* although semi-rigid, would not have been stable enough for brachiopod larvae to settle on, consequently giving the individual a buffer zone, discouraging the larvae of other brachiopods that could have potentially impeded feeding from settling on or close to their shells.

## Conclusions

Determining the functional mechanism of morphological features in fossil taxa is a difficult task, especially if the same features are not directly comparable and not fully understood in extant taxa. The sheer size of the chaetae possessed by both *M. burgessensis* and *P. zenobia* suggests that they must have held an important functional significance. A protective sensory role has been one of the most commonly proposed hypotheses to explain brachiopod chaetae and there is some evidence from the Burgess Shale to suggest that chaetae acted in this way for *Micromitra* and *Paterina*. Brachiopods were prey items in the Burgess Shale and an early sensory warning system would have been beneficial for protection against predators and consequently a sensory role cannot be discounted. That said, it is not clear how effective as a sensory tool the chaetae would have been for predators such as *Ottoia* and *Sidneyia*, and there is a distinct lack of smaller scale durophagous predation to support the construction of such elaborate chaetal structures. It is also possible that rather than a pure sensory role, that the substantial increase in brachiopod body size caused by the possession of these long, semi-rigid chaetae impeded ingestion and was a deterrent against predators, such as *Ottoia*. The arrangement and spacing of chaetae in *M. burgessensis* and *P. zenobia* provides little support for the chaetae playing a sieving role to obstruct excessive intake of fine particles and the lack of chaetal clustering discounts the chaetae forming a siphon to assist in creating feeding currents. The soft, muddy Burgess Shale seafloor provided limited hard substrata for brachiopods to attach to and the need to settle and, once settled, retain the ability to feed efficiently would likely have been the primary focus of the individual. Creating a chitinous buffer zone to impede larvae of other brachiopods settling on or in the immediate vicinity increases the likelihood of unimpeded growth. The increased competition in response to the lack of suitable substrate in the environment could have driven the emergence of exaggerated adaptations resulting in extreme length of chaetae seen in brachiopods of the Burgess Shale community and represents a plausible interpretation presented herein. An alternative view that the chaetae of *M. burgessensis* chaetae evolved to mimic characteristics of the co-occurring unpalatable sponge, *Pirania muricata* to deter potential predators, remains speculative, however the remarkable morphological similarities provides a conceivable and justifiable hypothesis. Documentation of brachiopod unbiomineralized anatomy is paramount in our understanding of the evolution and ecology of one of the oldest animal phyla. The functional mechanisms of brachiopod chaetae proposed here delivers a new perspective on their role in early brachiopod taxa and is critical not only to our understanding of the ecology of early brachiopod assemblages, but also the benthic dynamics of early Cambrian marine ecosystems.

## Methods

### Material

This study is based on 282 brachiopod specimens (208 specimens of *Micromitra* and 74 specimens of *Paterina*) from the Cambrian (Series 3, Stage 5) Burgess Shale Formation, Yoho National Park, Canada. The examined specimens (Additional file 1) are housed at the Royal Ontario Museum (acronym: ROM), the National Museum of Natural History, Smithsonian Institution (acronym: USNM) and a small selection at the Geological Survey of Canada (acronym: GSC). Extant brachiopod genera (Figure 5) are housed at the Swedish Museum of Natural History (SMNH). The majority of specimens were collected *in situ* on Fossil Ridge in British Columbia, predominantly from the Greater Phyllopod Bed (WQ and BW) [99,100] and the Raymond Quarry. Some specimens of *M. burgessensis* and *P. zenobia* (99 specimens) were collected from talus material above and below the Walcott and Raymond quarries (WT and RT) and come from the Trilobite Beds (ST) and also potentially from the Emerald Lake Oncolite Member [120]. Specimens were photographed under normal and cross-polarized light and wet and dry conditions using a Canon EOS6D digital SLR camera. Scanning electron microscope (SEM) photographs of uncoated specimens (Figure 3) were undertaken using a Zeiss Supra 35 VP microscope.

## Additional file

Additional file 1:
**Table detailing the material studied, including locality information, collection number, presence or absence of chaetae and the attachment strategy exhibited by individual specimens.** Abbreviations: M – *Micromitra burgessensis*, P – *Paterina zenobia*, N – *Nisusia burgessensis*, A – *Acrothyra gregaria*, C – specimen preserved with identifiable chaetae. The specimens are housed in the Royal Ontario Museum (ROM), Toronto, Canada, the Smithsonian National Museum of Natural History (USNM), Washington DC and three specimens at the Geological Survey of Canada (GSC), Ottawa, Canada.
